# Mobility during the post-partum period and viraemia in women living with HIV in South Africa

**DOI:** 10.1093/inthealth/ihad001

**Published:** 2023-01-28

**Authors:** Jasantha Odayar, Tamsin K Phillips, Siti Kabanda, Thokozile R Malaba, Elton Mukonda, Nei-yuan Hsiao, Maia Lesosky, Landon Myer

**Affiliations:** Division of Epidemiology and Biostatistics, School of Public Health, University of Cape Town, Cape Town, South Africa; Division of Epidemiology and Biostatistics, School of Public Health, University of Cape Town, Cape Town, South Africa; Division of Epidemiology and Biostatistics, School of Public Health, University of Cape Town, Cape Town, South Africa; Division of Epidemiology and Biostatistics, School of Public Health, University of Cape Town, Cape Town, South Africa; Division of Epidemiology and Biostatistics, School of Public Health, University of Cape Town, Cape Town, South Africa; Division of Medical Virology, National Health Laboratory Service, University of Cape Town and Groote Schuur Hospital, Cape Town, South Africa; Division of Epidemiology and Biostatistics, School of Public Health, University of Cape Town, Cape Town, South Africa; Division of Epidemiology and Biostatistics, School of Public Health, University of Cape Town, Cape Town, South Africa

**Keywords:** adherence, antiretroviral therapy, mobility, post-partum, travel, viral load

## Abstract

**Background:**

We investigated the association between travel and viraemia in post-partum women with human immunodeficiency virus on antiretroviral therapy (ART).

**Methods:**

Data are from a trial of post-partum ART delivery strategies. Women who initiated ART during pregnancy, were clinically stable with a viral load (VL) <400 copies/ml and were <10 weeks post-partum were enrolled at a primary care antenatal clinic in Cape Town, South Africa. Study visits at 3, 6, 12, 18 and 24 months post-partum included questions about travel, defined as ≥1 night spent outside of the city, and VL testing. Generalised mixed effects models assessed the association between travel and subsequent VL ≥400 copies/ml.

**Results:**

Among 402 women (mean age 29 y, 35% born in the Western Cape), 69% reported one or more travel events over 24 months. Being born beyond the Western Cape (adjusted odds ratio [aOR] 2.03 [95% confidence interval {CI} 1.49 to 2.77]), duration post-partum in months (aOR 1.03 [95% CI 1.02 to 1.05]) and living with the child (aOR 0.60 [95% CI 0.38 to 0.93]) were associated with travel. In multivariable analyses, a travel event was associated with a 92% increase in the odds of a VL ≥400 copies/ml (aOR 1.92 [95% CI 1.19 to 3.10]).

**Conclusions:**

Interventions to support women on ART who travel are urgently required.

## Introduction

Human immunodeficiency virus (HIV) is a chronic condition that requires lifelong adherence to treatment and retention in care.^1^ South Africa has the largest HIV epidemic in the world, with an estimated 7.5 million people living with HIV in 2020, of whom almost two-thirds are women >15 y of age.^2^ While 78% of women living with HIV in South Africa are estimated to be on antiretroviral therapy (ART), there are ongoing concerns regarding disengagement from care.^3^ Risk of disengagement is particularly high in post-partum women, with less than two-thirds of women on ART retained in care at 24 months post-partum in one analysis.^4^,^5^ ART non-adherence contributes to HIV disease progression, virological failure, ART drug resistance and HIV transmission.^6^,^7^ The risk of HIV transmission continues throughout breastfeeding, and maintaining engagement in care in post-partum women is important to reduce vertical transmission during breastfeeding and improve maternal outcomes.^3^,^8^

Mobility is a critical factor affecting engagement in chronic care services.^9^,^10^ Population mobility may take many forms, including permanent relocation, circular mobility (involving movement back and forth between multiple residences) and episodic travel.^11^,^12^ Population mobility is high in sub-Saharan Africa, including South Africa. In South Africa, people move for multiple reasons, including employment, education and access to healthcare, and mobility is predominantly internal, with permanent or temporary relocation from rural to urban areas and frequent travel to homes in rural areas.^11^,^12^ Among women, mobility is increasing and is predominantly of a temporary and localised nature, which may be difficult to measure.^11–13^ Post-partum women in South Africa are highly mobile, with many travelling to their rural homes post-delivery.^5^,^14^,^15^

Mobile populations face numerous challenges to maintaining continuous HIV care. Mobility has been shown to impact ART adherence in general populations.^10^,^15–18^ Interruptions in medication supply may occur, particularly when trips are of longer duration,^9^,^10^ and mobile individuals may thus require access to care at multiple health facilities. However, transfer between facilities has been associated with an increased risk of viraemia in general adults and in post-partum women.^19^ Despite the risks of disengagement in mobile populations and evidence that travel occurs frequently immediately post-partum, there are few data on travel and viral load outcomes in the extended post-partum period. A better understanding of travel patterns and the association between travel, healthcare usage and HIV treatment outcomes over the extended post-partum period is required to inform and target possible interventions. We assessed the frequency of travel, predictors of travel, health facility attendance while travelling and the association between travel and viral load (VL) in a cohort of women on ART through 24 months post-partum.

## Methods

### Study design and participants

We hypothesized that travel among post-partum women may lead to non-adherence or treatment interruption and subsequent viraemia. To assess the association between travel and viraemia, we conducted a secondary analysis of data from a randomized controlled trial of differentiated care for post-partum ART delivery (Postpartum Adherence Clubs for Antiretroviral Therapy [PACART]; NCT03200054) in Cape Town, South Africa.^20^ The study was conducted at a large Community Health Centre (CHC) and associated Midwife Obstetric Unit (MOU) in Cape Town. This public-sector primary health care facility serves a population of approximately 350 000, which is predominantly of low socio-economic status.^21^ The MOU provides antenatal care (ANC), services to prevent vertical transmission of HIV, obstetric services and post-natal care to >4000 women annually. ANC uptake in the community is high (>95%), as is antenatal HIV prevalence, estimated at 30% in 2013. The CHC includes an ART clinic that provides HIV care to the general adult population.

Women attending the MOU post-delivery were screened for participation in the parent trial between January 2016 and December 2017 and were enrolled if they were ≥18 y of age, had started ART in the preceding pregnancy, were <10 weeks post-partum, had an HIV VL <400 copies/ml in the preceding 3 months and had no comorbidities requiring regular clinical follow-up. All women started tenofovir (TDF) 300 mg, lamivudine (3TC) 300 mg or emtricitabine (FTC) 200 mg, and efavirenz (EFV) 600 mg taken once daily as a fixed-dose combination (FDC) in pregnancy. Participants were randomized to either the primary healthcare (PHC) ART clinic (the control arm) or the local differentiated service delivery model (adherence clubs [ACs]; intervention arm). The ACs operate from a community hall located approximately 1 km from the antenatal clinic.^22^ Patients are provided with 1–2 months of treatment at PHC ART clinics, with 3 months supplied at the end-of-year holiday period. At ACs, 2 months of treatment are supplied at routine visits, with 4 months supplied at the end-of-year holidays. At the ACs, patients are allowed to send a representative, called a ‘buddy’, to collect their treatment at alternate visits. As part of adherence counselling, patients on ART are advised to inform the health facility before travelling in order to receive a referral letter and sufficient treatment.^23^ VL testing is done annually at the AC and every 6–12 months at the PHC ART clinic. Patients at the ACs with a VL ≥400 copies/ml are referred to the PHC ART clinic. At the ART clinic, together with adherence support, those with a VL of 400–1000 copies/ml have a repeat VL in 6 months and those with a VL ≥1000 copies/ml have a repeat VL in 2 months.

### Data sources

Consecutive post-partum women who initiated ART during pregnancy, were within 70 d post-partum and met local differentiated service delivery eligibility criteria (clinically stable with VL <400 copies/ml in the preceding 3 months) were included in the parent trial. After enrolment, follow-up visits in the primary trial were conducted at 3, 6, 12, 18 and 24 months post-partum. Blood samples for VL testing were drawn at enrolment (this was separate from the screening VL) and at each subsequent visit. All study VLs were conducted separate from VLs done as part of routine care. Face-to-face interviews completed at all visits included questions regarding medical history, whether women had travelled outside of the City of Cape Town for at least 1 night since the last visit and the duration of travel. Questions regarding travel were implemented several months into the study and not all women attending the 3- and 6-month visits were asked about travel. Other questions included whether they attended an ART service visit at a facility at their travel destination, whether there had been any treatment interruptions since the last visit, which were defined as 2-week periods without ART, and whether one or more doses had been missed in the 30 d preceding the visit. Regarding previous antiretroviral exposure, participants were asked whether they had taken short-course therapy for prevention of mother-to-child-transmission in a previous pregnancy and whether they had previously been started on a three-drug lifelong ART regimen. HIV VL testing was done by the National Health Laboratory Services using the RealTime HIV-1 assay (Abbott Molecular, Abbott Park, IL, USA).

### Analysis

Analyses were performed using STATA/BE version 17.0 (StataCorp, College Station, TX, USA). To assess the association between travel events and VL outcomes, regardless of duration, a travel event was defined as at least 1 night spent outside of the City of Cape Town. At the 3-, 6-, 12-, 18- and 24-month post-partum visits, the proportion of participants who had travelled since the previous visit and the number of times each participant travelled were counted. Frequencies and proportions, means with standard deviations (SDs) or medians with interquartile ranges (IQRs) were used to describe characteristics at enrolment in those who did and did not travel, and based on the number of travel events.

Logistic regression models using generalized estimating equations to account for repeated measures within individuals assessed predictors of travel and the association between travel and viraemia (VL ≥400 copies/ml) at the next study visit. This VL threshold was used because it is the value at which national guidelines recommend additional action by a clinician including a careful adherence assessment and consideration of an early repeat VL test.^24^ Characteristics identified a priori as potential confounders included age, socio-economic status, marital status, previous antiretroviral use, whether the baby lived with the mother and duration post-partum. Measures available for socio-economic status included type of housing, education and employment status. The intervention in the PACART trial was associated with a reduction in viraemia.^25^ We thus adjusted models assessing the association between travel and VL by PACART randomisation allocation. Results were reported as odds ratios (ORs) with 95% confidence intervals (CIs). A sensitivity analysis was done to assess an alternate VL threshold (VL ≥50 copies/ml) as the outcome. Additional analyses were conducted to assess the association between the duration of travel and VL outcomes. Travel was measured in days and was log transformed to obtain a normal distribution. Lastly, we conducted analyses stratified by randomisation allocation in the primary trial to assess for effect modification by mode of care delivery.

## Results

**Figure 1.**
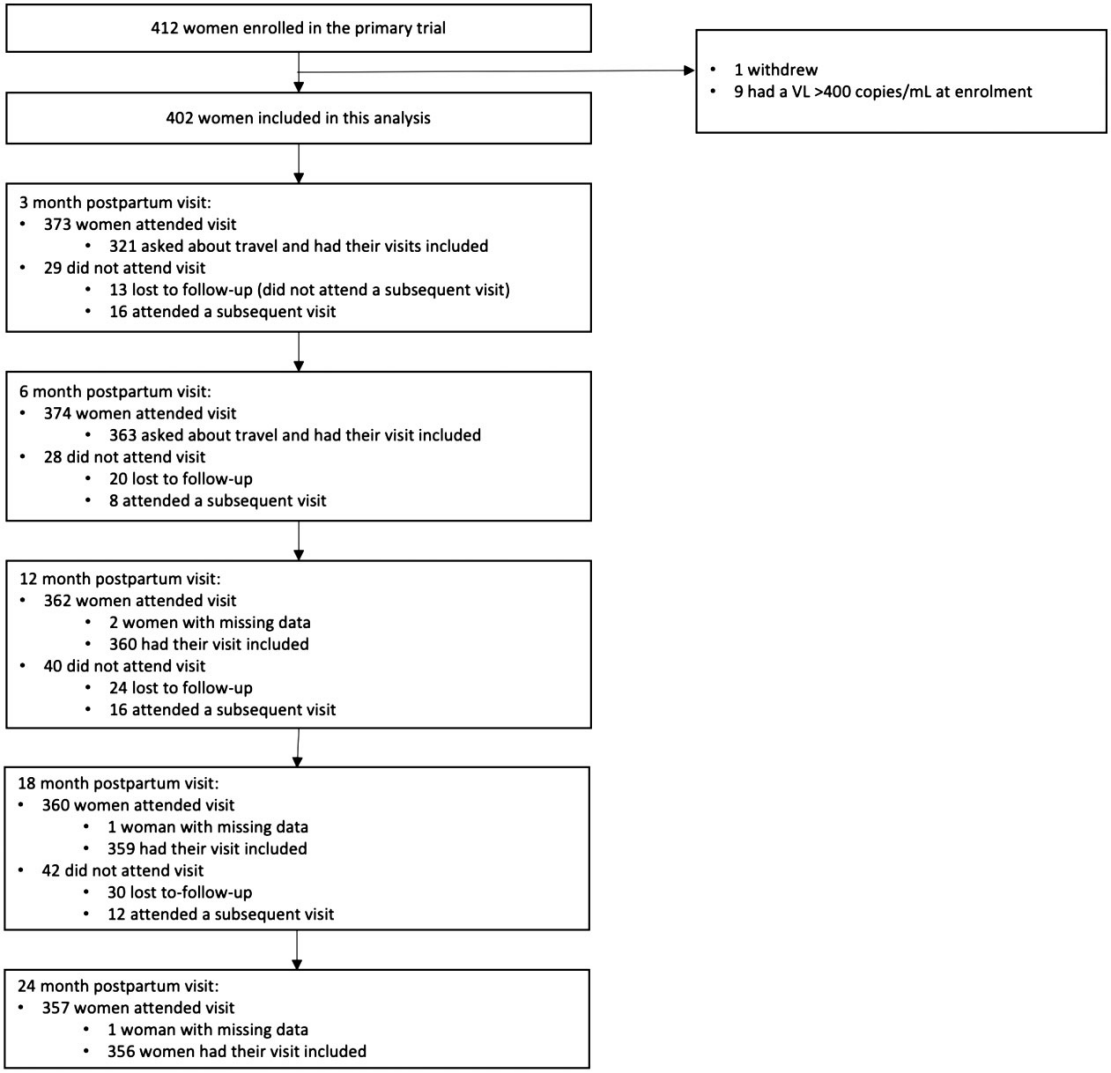
Inclusion of visits in the analysis.

Among the 412 women enrolled in the parent trial, 1 withdrew. In addition, the VL conducted at the enrolment study visit was ≥400 copies/ml in nine participants and they were excluded from further analysis (Figure 1). Of the remaining 402 women, mean age was 29 y (SD 5.2), 384 (96%) were born in South Africa and 139 (35%) were born in the Western Cape (Table 1). Overall, 169 women (42%) were married or cohabiting with a partner, 214 (53%) lived in informal housing and 396 (99%) had completed at least some high school. The median duration post-partum at enrolment was 10 d (IQR 6–20). Approximately one-quarter of enrolled women (n=105 [26%]) had a history of antiretroviral use (either ART or short-course prevention of vertical transmission) prior to the index pregnancy. All women had initiated ART in pregnancy and the median duration on ART at enrolment was 164 d (IQR 126–200).

**Table 1.** Enrolment characteristics of women included in the analysis, overall and by travel status

Characteristics	Total (N=402)	One or more travel events over 24 months post-partum (n=279)	No travel over 24 months post-partum (n=123)	p-Value	One travel event (n=124)	Two travel events (n=77)	Three or more travel events (n=78)	p-Value
Age (years), mean (SD)	29.2 (5.2)	29.3 (5.3)	28.9 (4.9)	0.5930	29.7 (5.3)	28.9 (5.0)	29.0 (5.8)	0.5613
Born in South Africa, n (%)	384 (95.5)	269 (96.4)	115 (93.5)	0.192	121 (97.6)	76 (98.7)	72 (92.3)	0.125
Born in the Western Cape, n (%)	139 (34.6)	74 (26.5)	65 (52.9)	<0.001	40 (32.3)	21 (27.3)	13 (16.7)	0.050
Completed any high school, n (%)	396 (98.5)	277 (99.3)	119 (96.8)	0.074	122 (98.4)	77 (100.0)	78 (100.0)	0.504
Currently employed and/or studying, n (%)	127 (31.6)	86 (30.8)	41 (33.3)	0.618	45 (36.3)	21 (27.3)	20 (25.6)	0.204
Live in informal housing, n (%)	214 (53.2)	169 (60.6)	45 (36.6)	<0.001	77 (62.1)	49 (63.6)	43 (55.1)	0.499
Married or cohabiting, n (%)	169 (42.0)	131 (47.0)	38 (30.9)	0.003	59 (47.6)	37 (48.1)	35(44.9)	0.908
Primiparous, n (%)	84 (20.9)	57 (20.4)	27 (22.0)	0.743	20 (16.1)	21 (27.3)	16 (20.5)	0.172
Missing, n (%)	1 (0.3)	1 (0.4)	0		1 (0.8)	0	0	
Delivered multiples in index pregnancy, n (%)	5 (1.2)	5 (1.8)	0 (0)	0.329	4 (3.2)	0 (0.0)	1 (1.3)	0.390
Previous triple-drug ART before index pregnancy, n (%)	47 (11.7)	31 (11.1)	16 (13.0)	0.514	18 (14.5)	7 (9.1)	6 (7.7)	0.248
Missing, n (%)	6 (2.0)	3 (1.1)	5 (4.1)		2 (1.6)	0	1 (1.3)	
Previous short-course PMTCT, n (%)	80 (19.9)	59 (21.2)	21 (17.1)	0.346	29 (23.4)	14 (18.2)	16 (20.5)	0.671
Any previous ARV exposure (triple-drug ART or PMTCT), n (%)	105 (26.1)	73 (26.2)	32 (26.0)	0.975	37 (29.8)	18 (23.4)	18 (23.1)	0.458
Infant tested HIV positive at birth, n (%)	0	0	0					
Duration on ART at randomisation (days), median (IQR)	163.5 (126–200)	168 (128.5–202)	154 (120–196)	0.0654	165 (120–199)	166.5 (136–197)	176 (146–206)	0.2573
Missing, n (%)	8 (2.0)	3 (1.1)	5 (4.1)		2 (1.6)	0	1 (1.3)	
Regimen initiated in pregnancy, n (%)								
TDF/XTC/EFV	391 (97.3)	273 (97.9)	118 (95.9)	0.322	122 (98.4)	75 (97.4)	76 (97.4)	0.815
Other	4 (1.0)	4 (1.4)	0		1 (0.8)	2 (2.6)	1 (1.3)	
Missing	7 (1.7)	2 (0.7)	5 (4.1)		1 (0.8)	0	1 (1.3)	
Time post-partum at randomisation (days), median (IQR)	10 (6–20)	10 (6–19)	12 (6–22)	0.1194	10 (6–24)	10 (7–16)	8 (5–17)	0.2596
Missed a dose in the last 30 days, n (%)	83 (20.7)	50 (17.9)	33 (26.8)	0.042	16 (12.9)	22 (28.6)	12 (15.4)	0.015
Randomised to ACs, n (%)	203 (50.5)	150 (53.8)	53 (43.1)	0.049	69 (55.7)	40 (52.0)	41 (52.6)	0.851
VL at randomisation <50 copies/ml, n (%)	355 (88.3)	242 (86.7)	113 (91.9)	0.140	107 (86.3)	67 (87.0)	68 (87.2)	0.980

ARV, antiretroviral; PMTCT, prevention of mother-to-child transmission; XTC, emtricitabine or lamivudine.

Of the 402 women who were included in the analysis 373 (93%), 374 (93%), 362 (90%), 360 (90%) and 357 (89%) attended study visits for the parent trial at 3, 6, 12, 18 and 24 months post-partum, respectively. As questions regarding travel were instituted a few months into the study, 321/373 (86%) and 363/374 (97%) women who attended the 3-month and 6-month post-partum visits, respectively, were asked travel-related questions. Further, two, one and one women attending the 12-, 18- and 24-month visits, respectively, had missing data regarding travel, leaving 360/362 (99%), 359/360 (99%) and 356/357 (99%) women who attended study visits with travel-related data at these visits.

A total of 279 (69%) women reported at least one travel event over the 24 months post-partum; of these, more than half (n=155 [56%]) travelled more than once. Compared with women who never travelled, a higher proportion of those who travelled lived in informal housing (61% vs 37%; p<0.001) at enrolment, were married or cohabiting (47% vs 31%; p=0.003) and were randomised to the AC arm at enrolment (54% vs 43%; p=0.049). A lower proportion of women who travelled were born in the Western Cape province (27% vs 53%; p<0.001).

A smaller proportion (48/321 [15%]) of women reported at least one travel event between enrolment and the 3-month post-partum visit compared with the post-partum visits at 3–6 (99/363 [27%]), 6–12 (114/360 [32%]), 12–18 (129/359 [36%]) and 18–24 months (114/356 [32%]) (Table 2). Travel was predominantly to one province, with >80% of women who reported travel at each visit travelling to the Eastern Cape province. The median duration of travel was 21 d. At all visits except for the 18-month post-partum visit, a higher proportion of participants who reported travelling did not have their baby living with them compared with those who did not report travelling. The proportion of women who attended an ART service visit at a health facility at their travel destination was 6% at 3 months post-partum and increased to 20% at 24 months post-partum. There were no clear differences in the proportions who reported a treatment interruption since the last visit or who reported missing a dose in the preceding 30 days between those who did and did not travel.

**Table 2.** Description of participants who travel and travel events by duration post-partum at which travel occurred

Characteristics	Enrolment to 3 months (n=321)			3–6 months (n=363)			6–12 months (n=360)			12–18 months (n=359)			18–24 months (n=356)		
	One or more travel events	No travel event	p-Value	One or more travel events	No travel event	p-Value	One or more travel events	No travel event	p-Value	One or more travel events	No travel event	p-Value	One or more travel events	No travel event	p-Value
Participants, n (%)	48 (15.0)	273 (85.1)		99 (27.3)	264 (72.7)		114 (31.7)	246 (68.3)		129 (35.9)	230 (64.1)		114 (32.0)	242 (68.0)	
Cumulative number who travelled	48			135			195			249			279		
Travel destination, n (%)															
Eastern Cape	41 (85.4)			83 (83.8)			101 (88.6)			112 (86.8)			105 (92.1)		
Western Cape	2 (4.2)			5 (5.1)			3 (2.6)			0			0 (0.0)		
Gauteng	2 (4.2)			4 (4.0)			5 (4.4)			6 (4.7)			1 (0.9)		
Other	3 (6.3)			7 (7.1)			5 (4.4)			11 (8.5)			9 (7.9)		
Total duration outside of Cape Town per participant (days), median (IQR)	21 (11.5–28)			21 (14–28)			21 (7–28)			21 (7–28)			21 (7–56)		
Missing, n (%)	0			1 (1.0)			0			5 (3.9)			4 (3.5)		
Infant not living with mother at start of visit interval, n (%)	4 (8.3)	2 (0.1)	0.005	13 (13.1)	11 (4.2)	0.002	20 (17.5)	15 (6.1)	0.001	20 (15.9)	36 (15.7)	0.956	34 (29.8)	34 (14.0)	<0.001
Missing, n (%)	0	2 (0.1)		0	0		2 (1.8)	1 (0.4)		3 (2.3)	0		3 (2.6)	4 (1.7)	
Attended a facility at travel destination, n (%)	3 (6.3)			13 (13.1)			11 (9.7)			17 (13.2)			23 (20.2)		
Missing, n (%)	0			0			0			1 (0.8)			0		
Treatment interruption (≥2 weeks without ART), n (%)	1 (2.1)	7 (2.6)	1.000	3 (3.0)	9 (3.4)	1.000	7 (6.1)	11 (4.5)	0.499	8 (6.2)	9 (3.9)	0.327	8 (7.0)	8 (2.9)	0.090
Missing, n (%)	0	0		0	1		0	0		0	0		0	0	
Missed dose in the last 30 d, n (%)	11 (22.9)	45 (16.5)	0.279	12 (12.1)	44 (16.7)	0.286	10 (8.8)	33 (13.4)	0.206	14 (10.9)	23 (10.0)	0.799	13 (11.4)	21 (8.7)	0.414
VL ≥50 copies/ml, n (%)	9 (18.8)	25 (9.2)	0.046	20 (20.2)	43 (16.3)	0.381	35 (30.7)	78 (31.7)	0.848	45 (34.9)	68 (29.6)	0.298	38 (33.3)	75 (31.0)	0.619
Missing, n (%)	0	0		0	0		0	0		0	0		1 (0.9)	0	
VL ≥400 copies/ml, n (%)	4 (8.3)	15 (5.5)	0.503	16 (16.2)	33 (12.5)	0.363	26 (22.8)	58 (23.6)	0.872	37 (28.7)	58 (25.2)	0.475	33 (28.9)	61 (25.2)	0.427
Missing, n (%)	0	0		0	0		0	0		0	0		1 (0.9)	0	
VL ≥1000 copies/ml, n (%)	4 (8.3)	14 (5.1)	0.324	13 (13.1)	32 (12.1)	0.795	22 (19.3)	52 (21.1)	0.688	32 (24.8)	51 (22.2)	0.570	30 (26.3)	55 (22.7)	0.432
Missing, n (%)	0	0		0	0		0	0		0	0		1 (0.9)	0	

In a univariate generalized mixed effects logistic regression model, randomisation allocation was not significantly associated with travel (OR 1.15 [95% CI 0.89 to 1.51]; Table 3). In adjusted analyses predicting travel, being born outside of the Western Cape province (adjusted odds ratio [aOR] 2.03 [95% CI 1.49 to 2.77]) and duration post-partum (aOR 1.03 [95% CI 1.02 to 1.05]) were associated with increased odds of a travel event. The relative odds of travel were reduced when the baby lived with the mother (aOR 0.60 [95% CI 0.38 to 0.93]). In a multivariable model predicting viraemia and adjusted for age, living in informal housing, being in a relationship or cohabiting, previous use of antiretrovirals (either ART or short-course prevention of mother-to-child transmission), randomization allocation, duration post-partum and whether or not they lived with the baby, the occurrence of a travel event since the last study visit was associated with a 92% increase in the relative odds of a VL ≥400 copies/ml (aOR 1.92 [95% CI 1.19 to 3.10]; Table 4). Using a VL ≥50 copies/ml as the outcome did not substantially alter the association (aOR 1.85 [95% CI 1.21 to 2.81]; Supplementary Table 1). When including only visit intervals in which the VL at the start of the interval was <400 copies/ml, the increased relative odds of a VL ≥400 copies/ml in those who travelled compared with those who did not persisted but was reduced (1.62 [95% CI 1.00 to 2.61]; Supplementary Table 2). There was a 24% increase in the relative odds of a VL ≥400 copies/ml associated with every 1 log increase in travel duration, but this was not statistically significant (aOR 1.24 [95% CI 0.85 to 1.81]; Supplementary Table 3). In analyses stratified by randomization allocation in the primary trial, the adjusted relative odds of a VL ≥400 copies/ml in those who travelled since the last visit compared with those who did not was higher in women randomised to the PHC clinics (aOR 2.35 [95% CI 1.12 to 4.91]) versus women randomised to the AC intervention (aOR 1.72 [95% CI 0.91 to 3.25]; Supplementary Table 4).

**Table 3.** Results of mixed effects logistic model for relative odds of travel event (n=388)

	Unadjusted		Adjusted	
Variables	OR	95% CI	OR	95% CI
Fixed effects (at enrolment)				
Born in South Africa	0.92	0.48 to 1.76		
Born outside of the Western Cape	2.30	1.73 to 3.05	2.03	1.49 to 2.77
Age at enrolment (years)	0.99	0.96 to 1.01	0.98	0.95 to 1.01
Lives in informal housing	1.60	1.23 to 2.09	1.19	0.90 to 1.57
Any previous ARV use (triple-drug ART or short-course PMTCT)	0.90	0.66 to 1.21		
Completed any high school	4.66	0.92 to 23.6		
Working and/or studying	0.87	0.65 to 1.16		
Cohabiting or married	1.38	1.06 to 1.81	1.30	0.99 to 1.71
Randomised to AC	1.15	0.89 to 1.51		
Time-varying fixed effects (at visit prior to travel event)				
VL ≥50 copies/ml	0.99	0.73 to 1.33		
Duration post-partum (months)	1.04	1.03 to 1.06	1.03	1.02 to 1.05
Living with baby	0.40	0.26 to 0.62	0.60	0.38 to 0.93

ARV: antiretroviral; PMTCT: prevention of mother-to-child transmission.

**Table 4.** Results of mixed effects logistic model for relative odds of VL ≥400 copies/ml (n=389)

	Unadjusted		Adjusted	
Variables	OR	95% CI	OR	95% CI
Fixed effects (at enrolment)				
Age (years)	0.96	0.90 to 1.01	0.91	0.83 to 1.00
Informal housing	0.60	0.32 to 1.09	0.50	0.21 to 1.21
Working and/or studying	0.59	0.30 to 1.17		
Married and/or cohabiting	0.40	0.22 to 0.75	0.31	0.12 to 0.78
Any previous ARV use (triple-drug ART or short-course PMTCT)	2.57	1.32 to 5.02	7.56	2.02 to 21.06
Duration between visits (months)	1.66	1.49 to 1.84		
Randomised to AC	0.54	0.29 to 0.99	0.46	0.20 to 1.09
Time-varying fixed effects (at visit prior to travel event)				
Travel since the last visit	2.15	1.41 to 3.26	1.92	1.19 to 3.10
Duration post-partum (months)	1.15	1.12 to 1.19	1.14	1.11 to 1.17
Living with baby	0.17	0.09 to 0.32	0.69	0.31 to 1.51

ARV: antiretroviral; PMTCT: prevention of mother-to-child transmission.

## Discussion

This analysis described travel and assessed VL outcomes post-travel among women on ART enrolled within 70 d post-partum and followed up through 24 months post-partum. Almost 70% of women reported at least one travel event over the study period. Women born outside of the province and those who did not live with their child were more likely to travel. In a multivariable model, travel was associated with a 92% increase in the relative odds of an elevated VL. Considering the documented number of women who travel and the associated risk of viraemia, improving outcomes in women who travel is vital if we are to improve treatment outcomes in post-partum women.

Mobility has been associated with non-adherence and non-retention in general adults living with HIV.^17–19^ Types of mobility and the effects of mobility on engagement in care may differ in different contexts and populations.^10^,^11^,^13^ Post-partum women living with HIV are at high risk of disengagement, yet data on mobility in this group are limited. In Kwazulu-Natal in South Africa, >20% of women relocated during pregnancy and the first year post-partum.^16^ Also in South Africa, travel was shown to occur frequently in the immediate post-partum period but was not assessed beyond this period.^14^ This analysis provides insights into travel up to 24 months post-partum in an urban area of South Africa with high levels of mobility and high numbers of women on ART. With the majority of women in this cohort travelling at least once over the study period, these findings emphasise the need for HIV care services that support mobile populations.

Travel was associated with viraemia regardless of duration. Research among general adults has shown that disruption of daily schedules and stigma among mobile patients who fear disclosing their HIV status by taking their medication around family or friends may lead to non-adherence.^10^,^26^,^27^ These factors may be relevant even for trips of short duration. The relative odds of travel increased with increasing duration post-partum, and this may be because women were enrolled at a median of 10 weeks post-partum, meaning that travel events prior to this were not included. Nevertheless, travel occurred throughout the 24 months post-partum, indicating that support for women who travel is required on an ongoing basis. In contrast to a study in Johannesburg, where post-partum women were found to travel throughout South Africa immediately post-delivery, travel in our setting was primarily to one province.^14^ This underscores the need to assess travel patterns in different settings to understand the locations where interventions may be required. We also found that women born outside of the province and those who did not live with their child were more likely to travel, which may allow targeting of interventions. In line with previous studies, we found a strong association between previous ART use and viraemia, possibly due to development of resistance or repeated non-adherence.^28^ Separation of mother and child has also previously been shown to be associated with an increased risk of viraemia in post-partum women on ART.^29^ One explanation for this finding is that women are motivated to take their treatment to stay healthy while with their children, but this motivation may decrease when separated. Our results suggest that travel may also play a role in the increased risk of viraemia in women separated from their children.

Provision of chronic care for mobile populations is an ongoing challenge.^9^,^30^ In the stratified analysis, the relative odds of viraemia in women who travelled compared with those who did not travel was higher among women randomised to the PHC clinics compared with the AC intervention arm of the primary trial. Possible reasons for this include increased social support at ACs and the increased spacing between AC visits, which may prevent women running out of treatment while travelling. Running out of treatment on longer trips has been cited as a reason for treatment interruption in qualitative studies.^26^,^31^ We found an increased relative odds of viraemia with increasing duration of travel; this was not statistically significant, but data on travel were collected every 3–6 months and recall bias may be present. Understanding how differentiated service delivery models such as ACs may support mobile populations requiring chronic care is a potential important avenue for further research. Patients who run out of treatment require access to health facilities at the travel destination.^26^ Based on self-reports, the proportion of women who travelled and attended a health facility at their destination in this analysis was low—4% at 3 months post-partum, increasing to 20% at 24 months. This could mean that women plan their trips to return before their medication runs out, they inform their original clinics of their travel plans and obtain sufficient treatment for the duration of their trip^14^ or they run out of treatment without attending a clinic at their travel destination. Numerous barriers to attending health facilities when away from home have been described, including stigma, negative interactions with healthcare workers and having to retell their history to healthcare workers,^9^ and transferring between health facilities has been associated with viraemia in general adults.^32^ Based on this, further investigation into access to care at travel destinations should be considered.

The strengths of this analysis include the 24-month duration of follow-up and the use of VL as an outcome measure, which provides an objective measure of adherence. Limitations include that travel data were collected every 3–6 months and recall bias may be present and data on engagement in care at the travel site and treatment interruptions were self-reported. In addition, we were unable to ascertain the duration between travel and VL assessment and we did not have data on reasons for travel, which may be associated with adherence.^33^ While follow-up rates in the study were high, loss to follow-up (LTFU) may bias results, as mobility may be associated with LTFU status and viraemia. This may have led to an underestimation of the number of travel events and of the association between travel and viraemia.

In summary, approximately 70% of women travelled one or more times through 24 months post-partum and travel was independently associated with an elevated VL. Interventions to support post-partum women on ART who travel are urgently required to improve treatment outcomes.

## Supplementary Material

ihad001_Supplemental_File

## Data Availability

Data are available from authors upon reasonable request.
