# Supplementary material for: Mobility during the post-partum period and viraemia in women living with HIV in South Africa
Source: Int Health. 2023 Jan 28;15(6):692–701. doi: 10.1093/inthealth/ihad001 (PMC10629960; doi:10.1093/inthealth/ihad001)
Supplement: ihad001_Supplemental_File [file ihad001_supplemental_file.docx]

| **Supplementary Table 1: Results of mixed effects logistic model for relative odds of VL >50 copies/mL adjusted for all presented variables** | | | |
| --- | --- | --- | --- |
|  | | OR | 95% CI |
| **Fixed effects (at enrolment)** | |  |  |
|  | Age (years) | 0.94 | 0.87–1.01 |
|  | Informal housing | 0.57 | 0.27–1.23 |
|  | Married and/or cohabiting | 0.37 | 0.17–0.82 |
|  | Any previous ART use | 3.57 | 1.49–8.55 |
|  | Randomised to AC | 0.43 | 0.21–0.92 |
| **Time-varying fixed effects (at visit prior to travel event)** | |  |  |
|  | Travel since the last visit | 1.85 | 1.21–2.81 |
|  | Duration postpartum (months) | 1.11 | 1.09–1.14 |
|  | Living with baby | 0.75 | 0.38–1.48 |
| *Abbreviations: AC, adherence club; ART, antiretroviral therapy; CI, confidence interval; OR, odds ratio; VL, viral load.* | | | |

| **Supplementary table 2: Results of mixed effects logistic model for relative odds of VL >400 copies/mL restricted to visit intervals in which the VL at the start of the interval is <400 copies/mL (n=383 participants with 1,468 intervals)** | | | |
| --- | --- | --- | --- |
|  | | OR | 95% CI |
| **Fixed effects (at enrolment)** | |  |  |
|  | Age (years) | 0.93 | 0.88–0.98 |
|  | Informal housing | 0.66 | 0.38–1.13 |
|  | Married and/or cohabiting | 0.54 | 0.30–0.97 |
|  | Any previous ARV use (triple-drug ART or short-course PMTCT) | 2.99 | 1.59–5.62 |
|  | Randomised to AC | 0.58 | 0.34–0.97 |
| **Time-varying fixed effects (at visit prior to travel event)** | |  |  |
|  | Travel since the last visit | 1.62 | 1.00–2.61 |
|  | Duration postpartum (months) | 1.04 | 1.00–1.07 |
|  | Living with baby | 0.62 | 0.30 – 1.28 |
| *Abbreviations: AC, adherence club; ART, antiretroviral therapy; ARV, antiretroviral; CI, confidence interval; OR, odds ratio; PMTCT: prevention of mother-to-child transmission; VL, viral load.* | | | |

| **Supplementary Table 3: Results of mixed effects logistic model for relative odds of VL >400 copies/mL with log transformed travel duration in days** | | | |
| --- | --- | --- | --- |
|  | | Adjusted OR | 95% CI |
| **Fixed effects (at enrolment)** | |  |  |
|  | Age (years) | 0.96 | 0.86–1.08 |
|  | Informal housing | 0.88 | 0.26–2.98 |
|  | Married and/or cohabiting | 0.18 | 0.05–0.67 |
|  | Any previous ART use | 7.61 | 1.80–32.17 |
|  | Randomised to AC | 0.54 | 0.17–1.74 |
| **Time-varying fixed effects (at visit prior to travel event)** | |  |  |
|  | Log travel duration since the last visit in days | 1.24 | 0.85–1.81 |
|  | Duration postpartum (months) | 1.12 | 1.06–1.19 |
|  | Living with baby | 0.86 | 0.25–2.98 |
| *Abbreviations: AC, adherence club; ART, antiretroviral therapy; CI, confidence interval; OR, odds ratio; VL, viral load.* | | | |

| **Supplementary table 4: Results of mixed effects logistic model for relative odds of VL >400 copies/mL stratified by randomisation allocation** | | | | | |
| --- | --- | --- | --- | --- | --- |
|  | | Randomised to ACs | | Randomised to PHCs | |
|  | | OR | 95% CI | OR | 95% CI |
| **Fixed effects (at enrolment)** | |  |  |  |  |
|  | Age (years) | 0.84 | 0.75 – 0.95 | 1.00 | 0.87 – 1.14 |
|  | Informal housing | 0.28 | 0.09 – 0.88 | 1.16 | 0.29 – 4.73 |
|  | Married and/or cohabiting | 0.49 | 0.15 – 1.62 | 0.16 | 0.04 – 0.71 |
|  | Any previous ARV use (triple-drug ART or short-course PMTCT) | 7.66 | 1.96 – 29.9 | 9.52 | 1.99 – 45.48 |
| **Time-varying fixed effects (at visit prior to travel event)** | |  |  |  |  |
|  | Travel since the last visit | 1.72 | 0.91 – 3.25 | 2.35 | 1.12 – 4.91 |
|  | Duration postpartum (months) | 1.13 | 1.09 – 1.17 | 1.17 | 1.11 – 1.22 |
|  | Living with baby | 0.58 | 0.19 – 1.77 | 0.84 | 0.27 – 2.58 |
| *Abbreviations: AC, adherence club; ART, antiretroviral therapy; ARV, antiretroviral; CI, confidence interval; OR, odds ratio; PHC: primary health care; PMTCT: prevention of mother-to-child transmission; VL, viral load.* | | | | | |
